# An annual time series of weekly size-resolved aerosol properties in the megacity of Metro Manila, Philippines

**DOI:** 10.1038/s41597-020-0466-y

**Published:** 2020-04-29

**Authors:** Connor Stahl, Melliza Templonuevo Cruz, Paola Angela Bañaga, Grace Betito, Rachel A. Braun, Mojtaba Azadi Aghdam, Maria Obiminda Cambaliza, Genevieve Rose Lorenzo, Alexander B. MacDonald, Preciosa Corazon Pabroa, John Robin Yee, James Bernard Simpas, Armin Sorooshian

**Affiliations:** 1 Department of Chemical and Environmental Engineering, University of Arizona, Tucson, Arizona USA; 2 Manila Observatory, Quezon City, 1108 Philippines; 3 Institute of Environmental Science and Meteorology, University of the Philippines, Diliman, Quezon City, 1101 Philippines; 4 Department of Physics, School of Science and Engineering, Ateneo de Manila University, Quezon City, 1108 Philippines; 5 Department of Hydrology and Atmospheric Sciences, University of Arizona, Tucson, Arizona USA; 6 Department of Science and Technology, Philippine Nuclear Research Institute, Commonwealth Avenue, Diliman, Quezon City, 1101 Philippines

**Keywords:** Atmospheric chemistry, Atmospheric chemistry

## Abstract

Size-resolved aerosol samples were collected in Metro Manila between July 2018 and October 2019. Two Micro-Orifice Uniform Deposit Impactors (MOUDI) were deployed at Manila Observatory in Quezon City, Metro Manila with samples collected on a weekly basis for water-soluble speciation and mass quantification. Additional sets were collected for gravimetric and black carbon analysis, including during special events such as holidays. The unique aspect of the presented data is a year-long record with weekly frequency of size-resolved aerosol composition in a highly populated megacity where there is a lack of measurements. The data are suitable for research to understand the sources, evolution, and fate of atmospheric aerosols, as well as studies focusing on phenomena such as aerosol-cloud-precipitation-meteorology interactions, regional climate, boundary layer processes, and health effects. The dataset can be used to initialize, validate, and/or improve models and remote sensing algorithms.

## Abstract (summary)

**Table:** 

Measurement(s)	ion concentration • concentration of water-soluble element • particulate matter • size distribution • mass concentration of black carbon
Technology Type(s)	ion chromatography • inductively coupled plasma mass spectrometry • Micro-Orifice Uniform Deposit Impactors (MOUDI) • Multi-wavelength Absorption Black Carbon Instrument (MABI) • gravimetric analysis
Factor Type(s)	geographic location
Sample Characteristic - Location	National Capital Region

Machine-accessible metadata file describing the reported data: 10.6084/m9.figshare.12037197

## Background & Summary

The composition and size distribution of ambient particulate matter (PM) influence how particles impact air quality and public health^1^, climate^2^, the hydrological cycle^3^, and geochemical cycling of nutrients and contaminants^4^. Depending on particle size and composition, an inhaled particle can deposit in the extrathoracic (head), tracheobronchial (TB), or pulmonary (PUL) regions, which can have serious implications for health^5–8^. Similarly, size and composition of particles impact their radiative properties, ability to act as cloud condensation nuclei (CCN), and also the ability to be transported between regions.

Since seasonal changes in meteorology, transport pathways, and emissions can impact a given region, annual PM cycles are important to characterize. A summary of past long-term (> three-month period) size-resolved PM substrate-based sampling efforts are provided in Online-only Table 1. There are a scarcity of annual time series data with at least weekly frequency regardless of global region. Most substrate-based sampling efforts for size-fractionated PM cover periods of one to three months with a sample collection duration between 24 to 96 hours per set, which were not included in Online-only Table 1. Longer sampling periods for individual sets, reaching up to a week^9,10^, are required in regions with less pollution in order to achieve sufficiently high mass concentrations (i.e. above limits of detection) for targeted species. The difficulty in obtaining long-term records of size-resolved PM composition with high temporal frequency is largely due to the labor-intensive nature of such measurements, which include several pre-and post- sampling steps and subsequent chemical analyses.

**Table 1:** List of instruments deployed at Manila Observatory (MO) before and during CAMP^2^ Ex and the associated measurement parameters.

Instrument	Parameters
Aerosol Robotic Network (AERONET)	Aerosol optical depth (AOD), single-scatter albedo (SSA), absorption angstrom exponent (AAE), scattering angstrom exponent (SAE), and water vapor
Disdrometer	Droplet size and vertical velocity
Arctic High Spectral Resolution Lidar (AHSRL)	Backscatter coefficient, depolarization ratio, and backscatter ratio
DustTrak and (2) Tactical Air Samplers (TAS)	Real-time and 24-hour total PM_2.5_ mass concentration with chemical speciation
Solar Spectral Flux Radiometer (SSFR)	Shortwave and longwave radiance and irradiance
All-Sky Camera	Hemispheric sky imaging
Particle Soot Absorption Photometer (PSAP)	Black carbon absorption and concentration
Automated Weather Station (AWS)	Temperature, relative humidity, wind speed, wind direction, solar radiation, pressure, and precipitation
Davis Rotation Uniform-Cut Monitor (DRUM)	Size segregated elemental composition of PM
Electronic Beta Attenuation Monitor (e-BAM)	Real-time PM_2.5_ mass concentration
Kipp and Zonen CMP22 Pyranometer	Solar radiation (broadband irradiance)
Kipp and Zonen CGR4 Pyrgeometer	Solar radiation (infrared irradiance)
SPN1 Shadow Pyranometer	Solar radiation (shadow broadband irradiance)
SP1-F Narrowband Shadow Pyranometer	Solar radiation (shadow narrowband irradiance)

The megacity of Metro Manila in the Philippines consists of 16 cities containing approximately 12.88 million people, with a collective population density of about 20,800 km^−2^ ^11,12^. Quezon City, the location where sampling took place, is one of the most populated cities in the region, with a population of about 2.94 million people and a population density of approximately 17,000 km^−2^ ^12^, which is among the highest in the world. Metro Manila is an ideal location for examining locally produced urban anthropogenic PM often mixed with a host of other air masses of marine and continental origin that are transported over both short and long distances to Metro Manila^13^. One aspect that makes the PM in Metro Manila unique is that black carbon levels are among the highest in the world^11,14,15^. The elevated black carbon is mainly due to vehicular emissions, more specifically the jeepneys, large trucks, and outdated vehicles^11^. The Philippines serves as a representative southeastern Asian country in terms of high population density, rapid urbanization, outdated vehicle usage and technology, and more lenient air regulations^11^.

The goal of this work is to present a 16-month size-resolved PM dataset for Metro Manila, Philippines. The unique geographic position of Metro Manila coupled to the wide ranging meteorology and transport patterns makes this dataset highly valuable in terms of examining numerous topics related to PM physics and chemistry with general implications for other regions: (i) impacts of PM on regional climate, clouds, and monsoonal activity, (ii) PM removal via wet deposition, (iii) aqueous processing of PM, (iv) source apportionment, (v) effects on PM properties due to mixing of varying air masses, (vi) catalytic and destructive effects of metals on inorganic/organic species, (vii) impacts of extreme events (e.g., biomass burning, dust storms, fireworks, typhoons) on regional PM, and (viii) public health implications.

## Methods

### Field study description

The dataset presented is a 16-month, size-resolved, chemical characterization of PM as part of a pre-campaign initiative for the Cloud, Aerosol, and Monsoon Processes Philippines Experiment (CAMP^2^Ex) titled CAMP^2^Ex weatHEr and CompoSition Monitoring (CHECSM) study. The CHECSM campaign took place between July 2018 through October 2019, within which August through October 2019 coincides with the airborne component of CAMP^2^Ex.

### Study site description

**Fig. 1:**
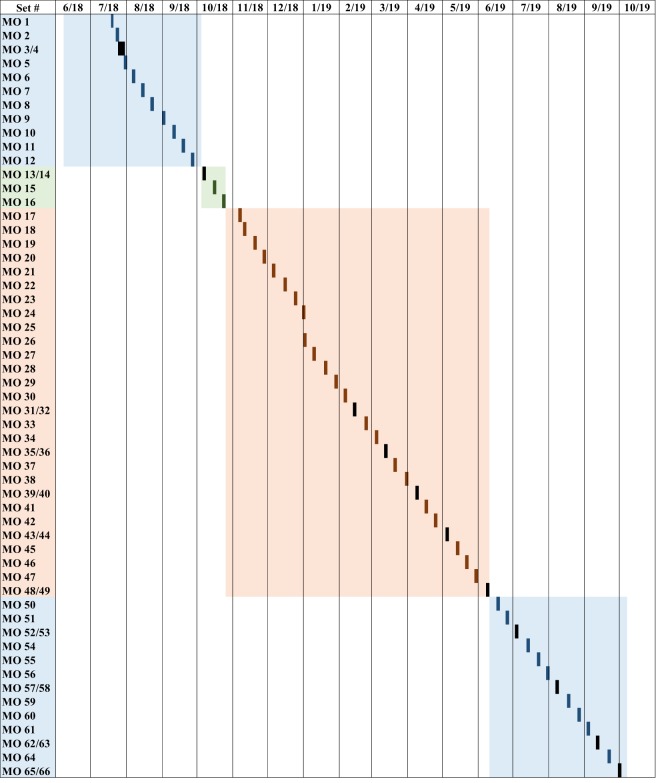
Timeline of size-resolved aerosol measurements at the Manila Observatory. Light blue boxes represent the southwest monsoon/wet seasons, the light green box represents the transitional period, and the orange box represents the northeast monsoon/dry season. Dark colored boxes represent MOUDI sampling periods and black boxes represent parallel MOUDI sampling periods.

The CHECSM study occurred at the Manila Observatory (MO; 14.64°N, 121.08°E) located at the Ateneo de Manila campus in Quezon City, Philippines. The site was segregated from surrounding urban areas, including a major roadway, by a grove of trees circling the campus. However, it was clearly impacted by local urban emissions and long-range transport based on results from the first six months of data collected^16–18^. Sampling took place on the 3^rd^ floor of the MO office building, which was approximately 85 m above sea level. Figure 1 shows a timeline of sampling, which occurred in four identified seasons: the 2018 southwest monsoon/wet season (18 June–4 October)^19,20^, a transitional period (5–25 October), the northeast monsoon/dry season (26 October 2018–10 June 2019)^21^, and the 2019 southwest monsoon/wet season (11 June–7 October)^22,23^. The southwest monsoon is characterized by relatively high temperatures, high humidity, frequent and heavy rainfall, and winds coming predominantly from the southwest. The northeast monsoon is characterized by moderate rainfall, low humidity, lower temperatures, and winds affecting the eastern side of the country. The characteristics of the monsoons listed above are general traits, but the major determining factor is rainfall. The measured temperature, humidity, and rainfall during sampling period collected at MO ranged from 25.4–30.2 and 24.2–30.9 °C, 59–94 and 54–85%, and 0–78.4 and 0–32.6 mm for the southwest and northeast monsoons, respectively, with average values of 27.6 and 27.7 °C, 72 and 64%, and 18.8 and 2.1 mm. Although the focus of this data descriptor is the size-resolved PM composition dataset, additional instrumentation co-located at MO during CHECSM is summarized in Table 1.

### Instrument description

Size-resolved PM was collected using a pair of Micro-Orifice Uniform Deposit Impactors II (MOUDI II 120 R, MSP Corporation, Marple *et al*.^24^) on Teflon substrate filters (PTFE membrane, 2 μm pores, 46.2 mm diameter, Whatman). The MOUDI-II is a 10-stage impactor with aerodynamic cutpoint diameters (D_*p*_) of 10, 5.6, 3.2, 1.8, 1.0, 0.56, 0.32, 0.18, 0.10, and 0.056 μm with a pre-impactor (> 18 μm) and an after-filter (< 0.056 μm). Refer to Table 2 for the associated stage numbers, collected diameter ranges, and cutpoint diameters. The instruments operated at a nominal flowrate of ~30 L min^−1^, with measured flowrates for each set reported in Table 3. Each stage, except for the pre-impactor and after-filter, continuously rotates to allow for uniform deposition of particles. Pressures for each stage were measured and recorded to ensure pressure drops were within acceptable ranges. An identical pair of MOUDIs were deployed for two reasons: (i) there would be no delay in sampling when a unit required maintenance, and (ii) simultaneous measurements allowed for additional analyses of collected PM.

**Table 2:** List of the stages and the respective collected diameter range and cutpoint diameters.

Stage #	Diameter Range (μm)	Cutpoint Diameter (μm)
1	> 18	18
2	18–10	10
3	10−5.6	5.6
4	5.6–3.2	3.2
5	3.2–1.8	1.8
6	1.8–1.0	1.0
7	1.0–0.56	0.56
8	0.56–0.32	0.32
9	0.32–0.18	0.18
10	0.18–0.10	0.10
11	0.10–0.056	0.056
12	< 0.056	< 0.056

**Table 3:** MOUDI sample set operating data. The table includes average flowrates, total sample run time, average operating temperature of the MOUDI cabinet, relative humidity (RH), and the days of the week sampling occurred. The start/end times varied between 13:00 and 15:00 local time for standard sets and 5:00 local time for dual gravimetric/IC sets. Sets with a label of (G) are gravimetric sets and the set labeled (AL) was collected for SEM analysis. All other sets were only measured with IC and/or ICP-QQQ.

Sample ID	Avg. Flow (L/min)	Run Time (hr)	Avg. Temp. (°C)	Avg. RH (%)	Days of the Week	Sample ID	Avg. Flow (L/min)	Run Time (hr)	Avg. Temp. (°C)	Avg. RH (%)	Days of the Week
MO1	29.6	24	30.5	59.0	Th-F	MO34	29.4	48	35.3	57.9	M-W
MO2	29.6	54	31.7	66.8	M-W	MO35 (G)	25.6	48	36.6	56.8	T-Th
MO3 (G)	28.6	119	34.9	69.0	W-M	MO36	29.3	48	39.9	56.8	T-Th
MO4	30.3	119	34.4	69.0	W-M	MO37	30.0	48	38.8	55.1	W-F
MO5	28.8	42	33.5	66.7	M-W	MO38	29.6	48	36.4	54.0	S-M
MO6	27.1	48	34.6	63.3	M-W	MO39 (G)	26.4	48	39.0	57.6	M-W
MO7	27.9	48	34.9	78.3	T-Th	MO40	29.6	48	41.4	57.6	M-W
MO8	29.0	48	35.7	78.2	W-F	MO41	29.1	48	38.7	57.7	T-Th
MO9	27.5	48	34.9	68.4	S-M	MO42	29.1	48	40.3	53.7	W-F
MO10	29.0	48	36.7	65.2	M-W	MO43 (G)	26.8	48	36.1	59.8	S-M
MO11	27.1	48	35.8	68.3	T-Th	MO44	28.6	48	37.0	59.8	S-M
MO12	27.5	48	37.0	70.9	W-F	MO45	28.7	48	37.3	61.8	M-W
MO13 (G)	29.8	48	35.1	73.1	S-M	MO46	28.7	48	39.0	72.2	T-Th
MO14	26.1	48	32.0	73.1	S-M	MO47	28.9	48	39.3	64.5	W-F
MO15	29.7	48	37.3	67.6	M-W	MO48	28.0	48	38.9	62.6	S-M
MO16	29.2	48	37.6	67.7	T-Th	MO49 (G)	25.5	48	38.1	62.6	S-M
MO17	30.0	48	36.5	60.6	W-F	MO50	28.8	48	39.2	64.4	M-W
MO18	29.5	48	36.7	61.9	S-M	MO51	27.8	50	36.2	77.1	T-Th
MO19	31.4	48	35.8	61.4	M-W	MO52 (G)	24.9	48	36.6	60.9	W-F
MO20	30.2	48	34.8	60.8	T-Th	MO53	26.9	48	38.8	60.9	W-F
MO21	30.5	48	34.8	72.0	W-F	MO54	28.8	48	36.8	66.4	S-M
MO22	29.6	48	32.7	78.5	S-M	MO55	28.8	48	38.0	75.4	M-W
MO23	26.4	48	29.7	81.8	M-W	MO56	26.7	48	35.0	76.1	T-Th
MO24	30.2	48	35.8	84.6	M-W	MO57	27.5	48	33.0	94.1	W-F
MO25 (AL)	N/A	2.75	N/A	N/A	M-T	MO58 (G)	24.5	48	33.4	94.1	W-F
MO26	24.1	48	35.0	77.2	T-Th	MO59	28.2	48	37.8	85.9	S-M
MO27	23.9	48	36.2	65.3	W-F	MO60	28.2	48	37.3	92.7	M-W
MO28	25.0	48	33.1	63.5	S-M	MO61	29.4	48	36.3	62.1	T-Th
MO29	29.5	48	34.5	63.3	M-W	MO62	27.8	48	36.5	77.0	W-F
MO30	29.8	48	34.4	60.7	T-Th	MO63 (G)	24.4	48	35.0	77.0	W-F
MO31	29.9	49	35.8	65.7	W-F	MO64	27.0	48	37.5	67.2	S-M
MO32 (G)	24.4	49	37.0	65.7	W-F	MO65	27.2	48	38.4	65.3	M-W
MO33	29.8	48	34.3	58.1	S-M	MO66 (G)	23.9	48	37.7	57.9	M-W

MOUDI sets were collected weekly over a 48-hour period with the exception of sets MO1, MO2, MO3/4, MO5, MO31/32, and MO51, which were collected for 24, 54, 119, 42, 49, and 50 hours, respectively. MOUDI sets labeled MO#/# refer to the sets that were simultaneously collected so that both chemical analysis and gravimetric analysis could be performed. A total of 66 sets were collected; 11 of the sets were collected using the simultaneous sampling approach, 54 of the sets were analyzed using ion chromatography (IC; Thermo Scientific Dionex ICS-2100 system), 47 of the sets were also analyzed using triple quadrupole inductively coupled plasma mass spectrometry (ICP-QQQ; Agilent 8800 Series), and 1 set (MO25) was collected as a special microscopy set. Additional MOUDI sets were collected on aluminum substrates for microscopy analysis using a Scanning Electron Microscope (SEM); however, these sets are not included in the dataset presented here. For more information on these sets, please refer to Cruz *et al*.^16^.

The MOUDIs were set up in such a way to reduce both particle losses and blockage of the inlet. The inlet tubing connecting the MOUDI to ambient air was constructed of stainless steel. The tubing was bent meticulously with a large radius such that there were no kinks. The inlet of the tubing was oriented downwards to prevent water from entering the MOUDI. To further avoid debris from getting into the inlet, a funnel with a mesh covering was attached securely to the downward facing tube opening exposed to ambient air. The temperature differential between the outside air and the tubing was either negligible or the tubing was slightly warmer than the outside air, thus reducing the possibility of thermal deposition. As the average relative humidity measured onsite over the sampling period was approximately 68% ranging from 54–94% throughout the year, the diameters of sampled particles correspond to wet rather than dry diameters and particle bounce was not significant^25^. This is additionally supported by particle morphology characterization showing evidence of halo areas, indicative of the particles being saturated when impacting onto the substrates^26–28^, surrounding particles in both the fine and coarse size ranges^16,17^.

### Pre-Sampling processing

The Teflon substrates were prepared prior to use by soaking each substrate face for a minimum of 12 hours in ~7.6 cm of Milli-Q (18.2 MΩ-cm) water in a laminar flow hood and/or covered container. Once each substrate face was soaked, the substrates were removed and placed in methanol cleaned Petrislides (Millipore), which were left slightly open in a laminar flow hood to dry any water residue. Once the substrates were dry, the Petrislides were closed and sealed using Parafilm to ensure the substrates were devoid of any particles or gases that could deposit on them.

### Post-Sampling processing

**Fig. 2:**
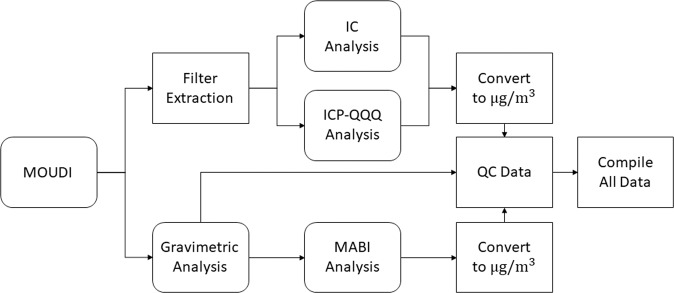
Flow chart of steps leading from MOUDI substrate collection to compilation of final data. The more commonly used single MOUDI sampling strategy follows only the top branch after “MOUDI” while the less frequent dual MOUDI sampling approach encompasses both the top and bottom branches. Rounded boxes represent instrument and analytical analyses steps while the standard boxes represent other processing steps.

Figure 2 summarizes the post-sampling process to reach the final dataset. After sampling was completed, substrates were first cut in half using ceramic scissors so one-half could be used for extraction in Milli-Q water and the other half could be stored in a freezer at −20 °C for future analyses. Ceramic scissors that were cleaned with methanol were used to cut the substrates in half in order to prevent contamination of heavy metals from other cutting instruments (e.g. metal scissors). The ceramic scissors were subsequently cleaned with methanol after each cut. Substrate extractions were performed using 8 mL of Milli-Q water (18.2 MΩ-cm) in cleaned 15 mL polypropylene centrifuge tubes that were sonicated for 30 minutes at 25–30 °C. Samples were extracted in this temperature range to ensure all targeted organics would solubilize. Additionally, during sampling, temperatures ranged from 28.7–45.7 °C; therefore, any volatile species were expected to be gone prior to the point of sampling and well before extractions took place. There have been other papers that performed similar extractions with temperatures up to 60 °C^29–33^. Sonicated solutions were then decanted into two different containers for analysis: (i) 0.5 mL polypropylene vial with a filter cap for analysis via IC, and (ii) a polypropylene centrifuge tube for analysis via ICP-QQQ. The remainder of the solutions were then stored in a refrigerator at 0 °C. Blank substrates were also processed in a similar manner to serve as background control samples. The motivation behind using water for extractions was owing to the importance of the results for health effects and toxicological studies, radiative effects, atmospheric residence time, nucleation efficiency, and bioavailability^34–39^.

### Ions

Cationic and anionic water-soluble PM speciation and quantification was conducted using a 2 mm IC system at a flowrate of 0.4 mL min^−1^. The cationic species measured were Na^+^, NH_4_^+^, Mg^2+^, Ca^2+^, dimethylamine (DMA), trimethylamine (TMA), and diethylamine (DEA) using an eluent of methanesulfonic acid. The anionic species measured were methanesulfonate (MSA), pyruvate, adipate, succinate, maleate, oxalate, phthalate, Cl^−^, NO_3_^−^, and SO_4_^2−^ using an eluent of potassium hydroxide (KOH). A 30-minute instrument method was used for both anion and cation columns with a 5-minute equilibration period giving a total of 35 minutes per sample. The columns used were the Dionex IonPac AS11-HC 250 mm and CS12A 250 mm models for anion and cation analysis, respectively. The suppressors used were a Dionex AERS 500e and a CERS 500e for anions and cations, respectively. For anions, the eluent started at 2 mM, ramped up to 8 mM from 0 to 20 minutes, and then ramped up from 8 to 28 mM from 20 to 30 minutes using a suppressor current of 28 mA. For cations, the eluent started at 5 mM, was isocratic from 0 to 13 minutes, ramped up from 5 to 18 mM from 13 to 16 minutes, and finally was isocratic at 18 mM from 16 to 30 minutes using a suppressor current of 22 mA. The recoveries, limits of detection (LOD), and limits of quantification (LOQ) for these species can be found in Table 4.

**Table 4:** Water-soluble species analyzed with their respective recoveries ± standard deviations (SD), limits of detection (LOD), and limits of quantification (LOQ) in aqueous concentration units. Species were quantified using IC (ions). LODs and LOQs in ppb are aqueous concentrations while LODs and LOQs in μg m^−3^ are air equivalent concentrations.

Ion	Recovery ± SD (%)	LOD (ppb)	LOQ (ppb)	LOD (μg m^−3^)	LOQ (μg m^−3^)
Adipate	101 ± 4	22.655	75.517	2.10E-03	6.99E-03
Ammonium	100 ± 17	42.434	141.447	3.93E-03	1.31E-02
Calcium	100 ± 5	45.229	150.763	4.19E-03	1.40E-02
Chloride	103 ± 7	2.144	7.147	1.99E-04	6.62E-04
DMA	100 ± 2	52.709	175.697	4.88E-03	1.63E-02
Magnesium	104 ± 8	36.925	123.083	3.42E-03	1.14E-02
Maleate	100 ± 3	6.970	23.233	6.45E-04	2.15E-03
MSA	102 ± 6	12.316	41.053	1.14E-03	3.80E-03
Nitrate	106 ± 12	8.917	29.723	8.26E-04	2.75E-03
Oxalate	100 ± 2	12.312	41.040	1.14E-03	3.80E-03
Phthalate	99 ± 2	20.685	68.950	1.92E-03	6.38E-03
Pyruvate	102 ± 6	63.754	212.513	5.90E-03	1.97E-02
Sodium	104 ± 8	43.476	144.920	4.03E-03	1.34E-02
Succinate	98 ± 9	11.046	36.820	1.02E-03	3.41E-03
Sulfate	101 ± 3	11.982	39.940	1.11E-03	3.70E-03
TMA & DEA	102 ± 4	315.164	1050.550	2.92E-02	9.73E-02

### Elements

Water-soluble elements were speciated and quantified using ICP-QQQ after being acidified in 2% nitric acid. The elements quantified were: Ag, Al, As, Ba, Cd, Co, Cr, Cs, Cu, Fe, Hf, K, Mn, Mo, Nb, Ni, Pb, Rb, Se, Sn, Sr, Ti, Tl, V, Y, Zn, and Zr. The recoveries, LOD, and LOQ for these species can be found in Table 5. For species that were measured by both IC and ICP-QQQ (Na, Mg, K, and Ca), duplications were not included in the dataset. IC measurements are provided for Na, Mg, and Ca, while ICP-QQQ measurements for K are provided due to potential contamination from the eluent (i.e., KOH) used in the IC. The exception to this is for sets MO57-MO65 where K from the IC was used due to lack of ICP-QQQ data.

**Table 5:** Same as Table 4 but species were quantified using ICP-QQQ (elements). Species marked with ‘—’ in their respective recovery and standard deviation columns were not measured for recovery purposes. LODs and LOQs in ppt are aqueous concentrations while LODs and LOQs in μg m^−3^ are air equivalent concentrations.

Element	Recovery ± SD (%)	LOD (ppt)	LOQ (ppt)	LOD (μg m^−3^)	LOQ (μg m^−3^)
Ag	100 ± 11	0.743	2.477	6.88E-08	2.29E-07
Al	96 ± 7	29.474	98.247	2.73E-06	9.10E-06
As	98 ± 10	7.945	26.483	7.36E-07	2.45E-06
Ba	97 ± 11	3.698	12.327	3.42E-07	1.14E-06
Cd	102 ± 11	4.194	13.980	3.88E-07	1.29E-06
Co	98 ± 8	0.722	2.407	6.69E-08	2.23E-07
Cr	97 ± 9	1.150	3.833	1.06E-07	3.55E-07
Cs	—	0.733	2.443	6.79E-08	2.26E-07
Cu	99 ± 8	1.127	3.757	1.04E-07	3.48E-07
Fe	97 ± 9	1.191	3.970	1.10E-07	3.68E-07
Hf	—	0.963	3.210	8.92E-08	2.97E-07
K	93 ± 18	10.480	34.933	9.70E-07	3.23E-06
Mn	97 ± 9	1.624	5.413	1.50E-07	5.01E-07
Mo	96 ± 11	2.258	7.527	2.09E-07	6.97E-07
Nb	—	0.522	1.740	4.83E-08	1.61E-07
Ni	97 ± 8	2.837	9.457	2.63E-07	8.76E-07
Pb	99 ± 8	0.503	1.677	4.66E-08	1.55E-07
Rb	—	1.566	5.220	1.45E-07	4.83E-07
Se	97 ± 10	82.393	274.643	7.63E-06	2.54E-05
Sn	97 ± 7	1.772	5.907	1.64E-07	5.47E-07
Sr	98 ± 9	1.102	3.673	1.02E-07	3.40E-07
Ti	101 ± 10	39.046	130.153	3.62E-06	1.21E-05
Tl	100 ± 8	0.383	1.277	3.55E-08	1.18E-07
V	95 ± 9	1.353	4.510	1.25E-07	4.18E-07
Y	—	0.523	1.743	4.84E-08	1.61E-07
Zn	96 ± 8	5.880	19.600	5.44E-07	1.81E-06
Zr	—	1.008	3.360	9.33E-08	3.11E-07

### Gravimetric

Gravimetric analysis was performed using a Sartorius ME5-F microbalance with a sensitivity of ±1 μg. The microbalance was located in a temperature and humidity-controlled room at 20–23 °C and 30–40% relative humidity with an airlock buffer. Clean substrates were weighed prior to sample collection and then weighed again after sampling ended. Before weighing took place, the filters were equilibrated in the room for at least 24 hours. After the equilibration time, each substrate was passed near a ^210^Po antistatic tip for 30 seconds to minimize measurement bias due to electrostatic charge at the surface of the substrate. Each substrate was weighed twice, once initially and then again 24 hours later. If the difference between these two weighings exceeded 10 μg, the substrate was weighed again 24 hours later and this process was repeated until the difference between weighings was less than 10 μg. The percent standard deviations for the weighings before and after sampling, respectively, were relatively negligible, with the highest being 0.005%. The PM mass was derived from the difference of the average substrate weight after sampling minus the average substrate weight before sampling. The standard deviation of the change in weight was then calculated for each PM substrate using the following error propagation equation:

1 $$S{D}_{d}=\sqrt{S{D}_{b}^{2}+S{D}_{a}^{2}}$$

where SD_d_ is the standard deviation of the difference, SD_b_ is the standard deviation of the substrate before sampling, and SD_a_ is the standard deviation of the substrate after sampling. The percent standard deviation across all stages and sets averaged out to be approximately 7%.

### Black carbon

The subsequently weighed substrates were then analyzed using a Multi-wavelength Absorption Black Carbon Instrument (MABI; Australian Nuclear Science and Technology Organisation). The MABI is an optical instrument used to quantify the mass concentration of black carbon by detecting the absorption for seven wavelengths (405, 465, 525, 639, 870, 940, and 1050 nm). The following equation was used to calculate black carbon concentration:

2 $$BC(ng\,{m}^{-3})=\frac{1{0}^{5}[A({cm}^{2})]}{[\varepsilon ({m}^{2}\,{g}^{-1})][V({m}^{3})]}ln\left[\frac{{I}_{{\rm{0}}}}{I}\right]$$

where ϵ is the mass absorption coefficient, A is the substrate collection area, V is the volume of air sampled, I_0_ is the measured light transmission through the blank substrate, and I is the measured light transmitted through the sample substrate. The mass absorption coefficient was provided in the MABI manual, collection area was retrieved based on impaction rings on the substrates, volume was calculated from flowrate and sample time, and light transmission was produced directly from the MABI.

### Data processing

IC and ICP-QQQ areas were converted to concentrations using Excel sheets formatted to use calibration curves, unit operations, and sampling information. The concentration files were then organized using an assortment of MATLAB codes to produce the data into the published state with gravimetric and black carbon data. Excel and MATLAB processing files are available upon request.

## Data Records

The dataset, located on figshare^40^, is in a specialized format used by the National Aeronautics and Space Administration (NASA) for field data, which is referred to as the ICARTT file format. The file name consists of the associated campaign, instrument used, sampling method, start date, revision number, and the end date. The format includes data notes in a README tab. These notes include the data principal investigator (PI), affiliated institution, mission name, the start date of data collection, the last data revision date, the number of variables, data flags, sampling platform and location, instrument information, brief description of the data, and revision log. The revision log states what revision the data is currently on and lists the previous revisions and their relative status. Additional tabs include the MOUDI stage cutpoints and size ranges, uncertainties and LODs, and the variable list and units. Data include ions, elements, gravimetric weights, and MABI measurements separated by stages in air equivalent mass concentrations (µg m^−3^). Note that the reported data are in air equivalent concentrations and typically are converted to dC/dlog D_p_ to properly look at the size distributions.

## Technical Validation

A number of experimental and data processing techniques were implemented to validate and better characterize the final data. The flowrate for each set was measured using a flowmeter (Mesa Labs Definer 220 series) three times both prior to and after each sampling period. The overall average of these values was used as the flowrate for each set. Additionally, pressures for each stage were measured at the beginning and end of sampling to ensure there was no significant change in the pressure drop. To keep the flowrate as close to 30 L min^−1^ as possible, the MOUDI nozzle plates were removed and cleaned regularly and especially if the flowrate dropped below 27 L min^−1^. The nozzle plates were cleaned by soaking the plates in either a methanol-water solution or in pure methanol for 24 hours or more. They were then removed and rinsed with methanol, followed by placement in a clean area to let the methanol evaporate. However, towards the ending of the sampling campaign the flowrate dropped to about 24 L min^−1^ and subsequent cleanings did not alleviate the problem. The issue was likely due to one or both of the lower nozzle plates (0.056 and 0.1 μm cutpoint diameters) being heavily clogged with the black carbon rich air and unable to be cleared without a more aggressive cleaning method.

Chromatogram peaks were automatically drawn by the IC and ICP-QQQ system software. However, for the IC only, the operator would view each chromatogram to adjust peak areas and add in missing species. LOD and LOQ were calculated using 3 S_a_b^−1^ and 10 S_a_b^−1^ methods, respectively, where S_a_ is the standard deviation of the response and b is the slope of the calibration curve^41^. Recoveries were calculated by taking the ratio of the mass of a specific measured species to the known amount of that species in that sample^42^. Recoveries for IC and ICP-QQQ were all above 93% with repeatability ranging from 2% to 18% (Tables 4 and 5). During data analysis, dC/dlog D_p_ plots (stages 2–11) were examined to ensure a normal distribution was obtained. If the first (stage 2) or last stage (stage 11) was higher than the next (stage 3) or previous stage (stage 10), respectively, then that stage was not considered for a particular set and viewed as having unreliable data. If a species was not measured for a stage, a value of −9999 was inputted. Similarly, if a species was below the LOD for a stage, a value of −8888 was inputted. A summary of the relative number of data points either missing (i.e., no ICP-QQQ data for last seven sets) or below the LOD for a specific species and stage can be seen in Table 6.

**Table 6:** Summary of the number of data points either missing (outside parenthesis) or below the LOD (inside parenthesis) for a given species and MOUDI stage. Note that there were a total of 54 possible data points for each species and stage. These counts exclude gravimetric and microscopy sets where chemical analysis was not performed. Refer to Table 3 for cutpoint diameters and diameter ranges.

Species	Stage 1	Stage 2	Stage 3	Stage 4	Stage 5	Stage 6	Stage 7	Stage 8	Stage 9	Stage 10	Stage 11	Stage 12
Ag	7(39)	7(42)	7(31)	7(32)	7(30)	7(30)	7(30)	7(29)	7(27)	7(33)	7(40)	7(40)
Al	7(5)	7(5)	7(0)	7(0)	7(0)	7(0)	7(0)	7(0)	7(1)	7(1)	7(23)	7(19)
As	7(41)	7(44)	7(39)	7(35)	7(34)	7(21)	7(4)	7(5)	7(5)	7(8)	7(37)	7(40)
Ba	7(5)	7(0)	7(0)	7(0)	7(0)	7(0)	7(0)	7(0)	7(0)	7(16)	7(33)	7(26)
Cd	7(36)	7(41)	7(35)	7(30)	7(27)	7(6)	7(0)	7(0)	7(0)	7(8)	7(33)	7(37)
Co	7(24)	7(31)	7(18)	7(11)	7(7)	7(11)	7(12)	7(12)	7(11)	7(25)	7(40)	7(35)
Cr	7(27)	7(27)	7(14)	7(14)	7(14)	7(14)	7(6)	7(12)	7(14)	7(14)	7(16)	7(14)
Cs	7(47)	7(46)	7(37)	7(23)	7(23)	7(11)	7(3)	7(0)	7(0)	7(2)	7(41)	7(46)
Cu	7(28)	7(28)	7(8)	7(8)	7(7)	7(7)	7(3)	7(2)	7(7)	7(8)	7(14)	7(13)
Fe	7(18)	7(21)	7(12)	7(4)	7(2)	7(4)	7(1)	7(2)	7(10)	7(16)	7(25)	7(17)
Hf	7(45)	7(47)	7(41)	7(34)	7(31)	7(40)	7(37)	7(41)	7(44)	7(42)	7(47)	7(47)
K	0(14)	0(12)	0(1)	0(0)	0(0)	0(0)	0(0)	0(0)	0(0)	0(0)	0(15)	0(15)
Mn	7(3)	7(1)	7(0)	7(0)	7(0)	7(0)	7(0)	7(0)	7(0)	7(0)	7(15)	7(15)
Mo	7(37)	7(40)	7(23)	7(12)	7(8)	7(6)	7(4)	7(4)	7(4)	7(8)	7(34)	7(38)
Nb	7(43)	7(44)	7(35)	7(28)	7(23)	7(30)	7(17)	7(25)	7(34)	7(44)	7(47)	7(41)
Ni	7(26)	7(26)	7(3)	7(3)	7(1)	7(1)	7(0)	7(0)	7(0)	7(2)	7(10)	7(17)
Pb	7(25)	7(24)	7(3)	7(0)	7(0)	7(0)	7(0)	7(0)	7(0)	7(0)	7(13)	7(13)
Rb	7(10)	7(8)	7(2)	7(0)	7(0)	7(0)	7(0)	7(0)	7(0)	7(0)	7(8)	7(12)
Se	7(37)	7(43)	7(27)	7(25)	7(14)	7(13)	7(11)	7(11)	7(16)	7(24)	7(39)	7(39)
Sn	7(38)	7(40)	7(35)	7(22)	7(18)	7(8)	7(4)	7(0)	7(1)	7(5)	7(36)	7(36)
Sr	7(1)	7(1)	7(0)	7(0)	7(0)	7(0)	7(0)	7(0)	7(7)	7(14)	7(23)	7(18)
Ti	7(12)	7(8)	7(0)	7(0)	7(0)	7(0)	7(2)	7(3)	7(2)	7(6)	7(24)	7(21)
Tl	15(35)	15(36)	15(34)	15(35)	15(34)	15(31)	15(20)	15(18)	15(21)	15(27)	15(34)	15(34)
V	7(41)	7(41)	7(33)	7(26)	7(23)	7(16)	7(4)	7(0)	7(1)	7(18)	7(40)	7(40)
Y	7(33)	7(35)	7(22)	7(15)	7(14)	7(22)	7(26)	7(32)	7(29)	7(33)	7(39)	7(40)
Zn	7(11)	7(13)	7(5)	7(0)	7(0)	7(0)	7(0)	7(0)	7(0)	7(0)	7(12)	7(12)
Zr	7(31)	7(36)	7(14)	7(5)	7(1)	7(7)	7(9)	7(21)	7(36)	7(30)	7(42)	7(34)
Adipate	4(39)	4(42)	4(22)	4(30)	4(30)	4(30)	4(32)	4(29)	4(25)	4(20)	4(39)	4(37)
Ammonium	0(28)	0(37)	0(11)	0(8)	0(6)	0(2)	0(1)	0(0)	0(0)	0(0)	0(10)	0(8)
Calcium	0(15)	0(14)	0(2)	0(0)	0(0)	0(0)	0(1)	0(5)	0(8)	0(15)	0(41)	0(33)
Chloride	0(11)	0(8)	0(1)	0(0)	0(0)	0(0)	0(0)	0(2)	0(2)	0(7)	0(39)	0(30)
DMA	0(52)	0(53)	0(43)	0(47)	0(47)	0(39)	0(27)	0(25)	0(29)	0(41)	0(46)	0(44)
Magnesium	0(12)	0(9)	0(0)	0(0)	0(0)	0(0)	0(0)	0(1)	0(1)	0(12)	0(34)	0(34)
Maleate	0(54)	0(54)	0(53)	0(49)	0(51)	0(48)	0(24)	0(16)	0(23)	0(52)	0(54)	0(53)
MSA	0(49)	0(51)	0(44)	0(42)	0(28)	0(22)	0(7)	0(4)	0(8)	0(11)	0(48)	0(49)
Nitrate	0(20)	0(19)	0(2)	0(0)	0(0)	0(0)	0(1)	0(1)	0(1)	0(5)	0(31)	0(21)
Oxalate	0(14)	0(13)	0(5)	0(0)	0(0)	0(0)	0(0)	0(0)	0(0)	0(0)	0(6)	0(20)
Phthalate	0(47)	0(51)	0(38)	0(19)	0(20)	0(31)	0(24)	0(24)	0(23)	0(42)	0(51)	0(44)
Pyruvate	0(48)	0(53)	0(50)	0(50)	0(48)	0(51)	0(51)	0(54)	0(53)	0(52)	0(54)	0(51)
Sodium	0(13)	0(12)	0(1)	0(0)	0(0)	0(0)	0(0)	0(0)	0(0)	0(1)	0(24)	0(19)
Succinate	0(48)	0(50)	0(43)	0(41)	0(38)	0(42)	0(35)	0(35)	0(41)	0(47)	0(52)	0(47)
Sulfate	0(11)	0(8)	0(0)	0(0)	0(0)	0(0)	0(0)	0(0)	0(0)	0(0)	0(1)	0(9)
TMA & DEA	0(54)	0(54)	0(54)	0(54)	0(54)	0(52)	0(39)	0(32)	0(34)	0(46)	0(53)	0(54)

**Fig. 3:**
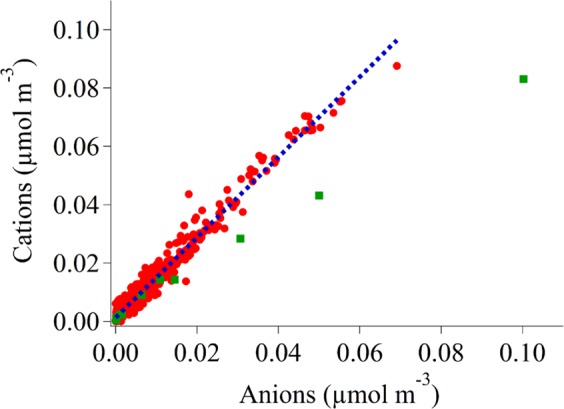
Charge balance plot for the cumulative MOUDI dataset using individual stages of all MOUDI sets. Red dots represent every stage of every set, with the exclusion of set 24, which is represented as green squares. The blue dashed line represents the line of best fit with a slope of 1.38 ± 0.01 and a R^2^ value of 0.97, excluding set 24, which was associated with New Year’s fireworks containing elevated anions and cationic transition metals.

A charge balance was also performed by converting each species to moles, multiplying by their respective charges, and then summing up all cations and all anions in a stage. It should be noted that only IC species, with the exception of K from ICP-QQQ, were used to measure the overall charge balances. The reasons for these are (i) the majority of the ICP-QQQ species are transition metals which have varying oxidation states and, without pH measurements, the proper charge cannot be assigned, and (ii) the majority of these species are very low in concentration and do not significantly affect the overall charge balances. All the stages were then plotted per set and a trend line was applied to test if there was a linear correlation. The charge balance R^2^ values in Table 7 reveal strong linear correlations (> 0.90), verifying that the data are valid. Additionally, Fig. 3 shows the overall charge balance for every set. All of the sets agree with the trend, with the exception of set 24, which can be seen deviating from the rest of the data. This set coincided with New Year’s fireworks, which produce a large amount of anionic species such as sulfate and nitrate as well as cationic metals, such as Fe and Cu. The combination of large anionic concentrations and the presence of cationic metals not included in the calculation lead to a charge balance slope below unity (i.e. more anions than cations).

**Table 7:** Slope and coefficient of determination (R^2^) of the water-soluble charge balance for each MOUDI set. Values above 1 indicate there is an anion deficit. Only IC species and K from ICP-QQQ are taken into consideration for the charge balance calculations.

Set #	Slope	R^2^	Set #	Slope	R^2^
MO1	0.89	0.92	MO31/32	1.19	0.94
MO2	1.42	0.99	MO33	1.26	0.95
MO3/4	1.21	1.00	MO34	1.43	0.98
MO5	1.36	0.99	MO35/36	1.36	1.00
MO6	1.32	0.98	MO37	1.37	0.94
MO7	1.36	0.99	MO38	1.29	0.95
MO8	1.36	1.00	MO39/40	1.50	0.97
MO9	1.26	0.99	MO41	1.50	0.99
MO10	1.35	1.00	MO42	1.46	0.96
MO11	1.26	0.84	MO43/44	1.44	1.00
MO12	1.33	0.99	MO45	1.35	1.00
MO13/14	1.29	1.00	MO46	1.47	1.00
MO15	1.30	0.99	MO47	1.60	0.99
MO16	1.42	0.98	MO48/49	1.70	0.97
MO17	1.39	0.96	MO50	1.94	0.99
MO18	1.33	0.98	MO51	1.43	0.94
MO19	1.47	0.98	MO52/53	1.63	0.94
MO20	1.29	0.95	MO54	1.46	1.00
MO21	1.30	0.97	MO55	1.38	0.98
MO22	1.27	0.97	MO56	1.57	0.94
MO23	1.27	0.94	MO57/58	1.24	0.96
MO24	0.82	1.00	MO59	1.45	1.00
MO26	1.46	0.91	MO60	1.29	0.96
MO27	1.55	1.00	MO61	1.39	0.97
MO28	1.17	0.97	MO62/63	1.24	0.97
MO29	1.50	0.87	MO64	1.36	1.00
MO30	1.66	0.91	MO65/66	1.44	0.99

## Usage Notes

The data provided can be used to conduct various studies to improve understanding of regional PM effects and implications. The dataset can be synchronized up with the other CHECSM instruments set up by the Air Quality Dynamics-Instrumentation and Technology Development (AQD-ITD) laboratory, the AErosol RObotic NETwork (AERONET) station^43^, and meteorological and precipitation chemistry data collected by MO (Table 2).

There are a host of previous (7 SouthEast Asian Studies (7-SEAS) 2010–2018; Biomass-burning Aerosols & Stratocumulus Environment: Lifecycles and Interactions Experiment (BASELInE) 2013–2015) and ongoing (CAMP^2^Ex) research activities in southeast Asia from which this dataset can provide additional context. The dataset also has relevance for all global regions in that process-level understanding can be improved using a dataset with such a wide range of pollution scenarios in one of the most polluted cities of the world with diverse meteorological characteristics.

A few papers have been produced using portions of this dataset already. Cruz *et al*.^16^ looked at size-resolved PM composition during the 2018 southwest monsoon season and conducted positive matrix factorization (PMF) to identify PM sources, which were attributed to aged PM, sea salt, combustion emissions, vehicular/resuspended dust, and waste processing emissions. Braun *et al*.^18^ presented case examples of long-range transport of PM from east and southeast Asia, such as biomass burning from the Maritime Continent and transport from continental East Asia. They also presented examples of different transport pathways of pollution to the study site which yielded concentration differences for species such as K, Rb, Ba, V, Pb, Mo, and Sn. AzadiAghdam *et al*.^17^ analyzed sea salt PM in Metro Manila and found that sea salt concentrations varied during the wet season and appeared to be contaminated by crustal and anthropogenic sources. Building off these limited examples using just a subset of the overall dataset, there are a significant number of topics that this dataset can be used to address, such as the following:

- Impacts of PM on regional climate, clouds, and monsoon activity by (i) comparing PM composition to other cities around the world with and without monsoon seasons, (ii) combining the dataset with meteorological data from satellites and models to understand influences on aerosol composition via mechanisms such as photochemical processing, and (iii) relating surface PM concentrations to AOD from AERONET and satellite sensors to examine the vertical nature of aerosol in the region as has been done in other regions (e.g. ref. ^44^).
- Removal of PM via wet deposition by looking at what species are most effectively scavenged using precipitation data (e.g. refs. ^45,46^).
- Aqueous processing of PM by looking at the changes of PM concentrations in the dry vs the wet season and additionally as a function of cloud coverage and aerosol liquid water amounts (e.g. refs. ^47,48^).
- Source apportionment of PM by (i) observing seasonal changes in emissions (e.g. ref. ^49^) and (ii) comparing the emission sources determined by techniques such as PMF for the 2018 southwest monsoon season versus the 2019 southwest monsoon season.
- Effects associated with mixing of varying air masses (e.g. ref. ^50^) by identifying (i) what air masses influence the city and during what times of year, (ii) if synergistic effects of mixing air masses can be seen year round, and (iii) if satellites and models that speciate aerosol can capture the behavior of mixing air masses in the region as reflected in the MOUDI data.
- Catalytic and destructive effects of metals on inorganic (e.g. refs. ^51–53^) and organic species (e.g. refs. ^54–56^).
- Impacts of extreme events on regional PM by examining (i) sets where holidays occurred (e.g. New Year’s) and (ii) sets influenced by typhoons, which have been shown to impact aerosol in the general region, such as was shown in previous studies in Taiwan^57^.
- Public health implications related to PM by examining the characteristic size distributions of species posing negative effects such as heavy metals and their general prevalence in Metro Manila.

## Online-only Table

**Online-only Table 1:** Summary of other “long-term” (> three-month period) papers published around the world that covered at least 3 seasons and had at least 10 sample sets using cascade impactors. The studies summarized provided most of the information (sampling period, sampling frequency, extraction type, and instrument). CH_2_Cl_2_ = dichloromethane, HNO_3_ = nitric acid, H_2_O_2_ = hydrogen peroxide, EtOH = ethanol, and MeOH = methanol.

Location	Sampling Period	Sampling Frequency	Extraction Type	Instrument	Reference
**Antarctica**					
Dome C, Antarctica	Jan 2006 - Dec 2007 Dec 2004 - Nov 2006	96-hour samples 168-hour samples	Water	Dekati (4-Stage) Andersen (8-Stage)	Becagli *et al*.^58^
**Asia**					
Bangkok, Thailand	Nov 1999 - Nov 2000	24-hour samples every 12 days	Acetonitrile	Andersen 235	Thongsanit *et al*.^59^
Guanzhou, China	Sum 2002 - Sum 2003	24-hour samples	CH_2_Cl_2_ Water	MOUDI 110	Tan *et al*.^60^
Jinan, China	Nov 2007 - Oct 2008	24-hour sampling every 2–3 days discontinuously	Water	MOUDI 110	Wang *et al*.^61^
Taiwan, China	Jan, Mar, Aug, Dec 2006, and Jul 2007	12-hour samples day/night alternating	Water	MOUDI Nano-MOUDI	Tsai *et al*.^62^
Characteristic North China	Sep 2009 - Aug 2010	24-hour samples bi weekly	Water	Anderson 20–800	Li *et al*.^63^
Dongying, China	Jan–Oct 2011	23.5-hour sampling periods	CH_2_Cl_2_/n-hexane (4:1)	MOUDI 110	Zhu *et al*.^64^
Lucknow, India	Oct–Dec 2011 Mar–May 2012	24-hour sample over 3 days	Aqua Regia	MOUDI-NR 110	Verma *et al*.^65^
Taiwan, China	Oct-Dec 2005 Jan, Mar, Aug, Dec 2006 Jul, Nov, Dec 2007	12-hour samples day/night	Water	MOUDI Nano-MOUDI	Tsai *et al*.^66^
Northwestern Beijing, China	Apr 22–30, 2007 Aug 2–14, 2007 Oct 24- Nov 7, 2007 Dec 13–23 2007	11.5/11-hour day/night (sample every day)	Water	MOUDI 110 MOUDI 100	Sun *et al*.^67^
Central Taiwan, China	Jan - Oct 2013	24-hour samples	HNO_3_/H_2_O_2_	MOUDI 110	Chen *et al*.^68^
Hong Kong, China	Aug - Sep 2011 Nov - Dec 2011 Feb - Mar 2012 May 2012	24-hour sampling	Water	MOUDI 110	Gao *et al*.^69^
Lhasa Station, China	Mar 2013 - Feb 2014	72-hour samples every 2 weeks	Water	Ambient 8-Stage Cascade Impactor	Wan *et al*.^70^
Marginal Seas of China (Bohai, North Yellow, South Yellow Seas)	Nov 2–19, 2012 Nov 6–25, 2013 Aug 18 - Sep 2015 Apr/May 3, 2015 Mar 31–Apr 2, 2015 May 4–5 2015	10–30 hour sample over 1–3 days	Water/EtOH	MOUDI	Yu *et al*.^71^
Beijing, China	Jul - Aug 2008 Dec 2009 - Feb 2010	23.5-hour samples	Water	Anderson 20–800	Liu *et al*.^72^
Kadapa, India	Mar 2013 - Feb 2015	72-hour samples	Water	Anderson 20–800	Begam *et al*.^73^
Shanghai, China	Apr 2015 Aug 2015 Oct 2015 Jan 2016	24-hour sampling daily	Water	MOUDI 110-R	Ding *et al*.^74^
Chongquing, China	Mar 2014 - Feb 2015	48-hour samples once a week	Water	Andersen 20–800	Li *et al*.^75^
Indoor sites Northern Taiwan, China	Jan 2014 - May 2017	24-hour samples	No extraction (size/weight distributions only)	MOUDI	Liu *et al*.^76^
Kofu, Japan	Feb 2014 - Feb 2015	720-hour samples	Water	Anderson AN–200	Matsumoto *et al*.^77^
Northern Urban Area Beijing, China	Jul 12–18, 2013 Jan 13–19, 2014 Jul 3–5, 2014 Oct 9–20, 2014 Jan 26–28 2015	11-hour samples day/night	Water	MOUDI 120	Su *et al*.^78^
Indoor sites Shanghai, China	Nov 26, 2015 - Feb 26, 2016 Apr 18 - Jun 29, 2016	72-hour samples	Water	MOUDI-II	Guo *et al*.^79^
**Europe**					
Belgium and Netherlands	Dec 1976 - Oct 1979	Variable	Benzene/MeOH	Sierra 230 Andersen 2000	Van Vaeck and Van Cauwenberghe^80^
Galway, Ireland; Tenerife, Canary Islands; United Kingdom	Jun 9 - Aug 5, 1996 Apr 29 - May 29, 1997 Jun 28 - Jul 23, 1997 Jun 14 - Jul 11, 1999 Jan 20 - Feb 17, 2000	24–93 hour samples	Water	MOUDI 110	Huang *et al*.^81^
Madrid, Spain	Apr 2004 - Apr 2005	24-hour sampling twice a week consecutively	CH_2_Cl_2_/Acetone (3:1)	CAV-A/HF	Pindado *et al*.^82^
Münster, Germany	Jan 2006 - Aug 2007	5–7.5 hour samples day/night	Water	Berner (5-Stage)	Gietl and Klemm^83^
Madrid, Spain	Feb 2007 - Feb 2008	7–12/6–8 hour day/night	Water	MOUDI 110 R	Plaza *et al*.^84^
Ljubljana, Slovenia	Dec 2014 - Nov 2015	48–72 hour samples	Water	Berner LPI	Frka *et al*.^85^
Mestre-Venice, Italy	Mar - May 2016	160-hours	Water	MOUDI-II 120	Barbaro *et al*.^9^
**North America**					
Mexico City, Mexico	Dec 2000 - Oct 2001	24-hour samples	Phthalic acid/acetone HNO_3_/water:MeOH	MOUDI 100	Moya *et al*.^86^
Claremont, California, US	Oct 2001 - Jul 2002	24-hour sampling once a week	CH_2_Cl_2_ Water	MOUDI 110	Miguel *et al*.^87^
Los Angeles, California, US	Dec 2000 - Jan 2001 Feb - Sep 2001 Sep 2001 - Aug 2002 Sep 2002 - Sep 2003	24-hour samples per week	Not reported	MOUDI 110	Sardar *et al*.^88^
Milwaukee, Wisconsin, US	July 2000 - Jan 2001	4–8 hour sampling period	Water	MOUDI	Lough *et al*.^89^
Gainesville, Florida, US	Feb - Dec 2000 Dec 2000 - Aug 2001	24-hour sampling	No extraction (carbon analysis only)	MOUDI 100	Chuaybamroong *et al*.^90^
Eastern Canada	Nov 2001 - Mar 2005	6–152 hour sampling	Water	MOUDI 110	Zhang *et al*.^91^
South America					
São Paulo, Brazil	Sep 2010 - Feb 2012	24-hour samples	CH_2_ Cl_2_/MeOH (4:1)	6-Stage Cascade Impactor	Urban *et al*.^92^
